# Addressing negative psychosocial factors linked to severe injury in professional rugby players: An introduction to a group psychotherapy approach

**DOI:** 10.17159/2078-516X/2020/v32i1a8505

**Published:** 2020-01-01

**Authors:** T M Hall, J A Botha, J S Patricios

**Affiliations:** 1 Counselling Psychologist, Private Practice, Kenilworth, Western Cape, South Africa; 2 Clinical Psychologist, Private Practice, Netcare Waterfalls, Johannesburg, South Africa; 3 Sport and Exercise Medicine Physician, Wits Sport and Health (WiSH), Faculty of Health Sciences, University of the Witwatersrand, Johannesburg

**Keywords:** psychosocial sequelae, mental health, rugby, injury

## Abstract

**Background:**

Negative psychosocial sequelae of severe rugby injury (SRI) in professional rugby players are well documented. Unaddressed, these issues can leave players vulnerable to persistent common mental disorders (CMD) and negatively affect injury recovery processes.

**Objective:**

To introduce a psychotherapeutic group intervention aimed at addressing negative psychosocial sequelae linked to SRI in professional rugby player cohorts.

**Methods:**

Literature aimed at clarifying the potential efficacy of an integrative group therapy model, the Recovery Mastery Group (RMG), is discussed after which component parts of the intervention are presented.

**Case illustration:**

A case illustration is presented comprising examples of how the RMG framework addressed psychosocial recovery issues in a professional South African rugby team during 2019.

**Conclusion:**

The proposed Recovery Mastery Group (RMG) is presented as a cost- and time- effective psychotherapeutic intervention that integrates well-researched psychotherapeutic techniques. The RMG appears able to address multiple facets of psychosocial injury recovery, while possibly offering protection from the onset of CMD. This introduction to the RMG can be a forerunner of similar research across larger cohorts, in different team sports, to determine wider therapeutic intervention efficacy.

Professional rugby union (rugby) has become a viable means of earning a living for many players. However, club salary caps, as well as international limitations on squad numbers, are escalating the need for administrators to protect existing contracted players from adverse factors associated with the professional game, including injuries and CMD.^[1]^

High rates of severe rugby injury (SRI) (i.e. > 28 days recovery) found in professional rugby can be costly to teams and to individual players physically, financially, and psychosocially.^[2]^ A growing body of literature outlines negative psychosocial sequelae of SRI that seem to be experienced in a stage-wise progression similar to the effects of a trauma experience.^[3,4,5]^

Documented negative psychosocial sequelae of SRI include feelings of fear, grief, loss, stress, and trauma reactions,^[6,7]^ as well as the added risk for the onset of CMD (e.g. distress, anxiety, depression, and sleep disturbance).^[1]^ Regarding CMD, Gouttebarge et al.^[1]^ found that the prevalence of anxiety and depression in the international elite rugby population was slightly higher than in general occupational populations. These authors suggested that severe time-loss musculoskeletal injuries are contributing stressors linked to higher rates of CMD onset. Furthermore, it stands to reason that psychosocial SRI reactions and the onset of CMD can affect a player’s physical injury recovery, quality of life and his/her ability to recover playing form.^[1,6,7,8]^

Recently, multiple researchers have suggested that more needs to be done in both understanding and addressing well-documented negative psychosocial sequelae of SRI that seem to be a common risk factor in the onset of CMD within the professional rugby population.^[1,6]^

## A multidimensional approach to SRI recovery

When attempting to understand SRI, the recovery experience, and how best to create effective recovery protocols, it is appropriate to consider multiple dimensions of this experience. Chang et al.^[9]^ in their position statement on mental health issues and psychological factors in athletes suggested that severe injuries are a contributing factor to mental health disorders in the elite athlete population. These authors advised that sport physicians, along with other care providers, when medically intervening with elite athletes, should be aware of relevant psychological, cultural, and environmental influences. Gouttebarge et al.^[1]^ also suggested that psychological attention should be included in the medical care of professional rugby players.

Hall^[3]^, drawing from the biopsychosocial model of sports injury rehabilitation processes,^[10]^ suggested that SRI affects the individual professional rugby player across biological, psychological and social dimensions of their life-world. The biopsychosocial model of sports injury rehabilitation considers seven dimensional influences: injury characteristics, socio-demographic factors, biological factors, psychological factors, social and contextual factors, intermediate biopsychosocial outcomes, and sports injury rehabilitation outcomes.^[10]^ Hence, the design of a psychotherapeutic intervention aimed at lessening the potential harmful effects of SRI should consider as many dimensions of the injury experience as possible. For the purposes of this paper, however, focus is drawn to rugby professional socio-cultural and psychosocial developmental factors taken into account in the design of the RMG.

## Socio-cultural factors

The professional rugby environment is generally one of routine, rituals and to a degree, tribal behaviour.^[11]^ Players live and work within a somewhat closed system that is potentially sceptical of outsiders, such as psychotherapists. A documented lack of psychotherapeutic referral networks, time and financial constraints,^[6]^ and a paucity of psychoeducation around psychosocial expectations of injury recovery,^[1,3,6]^ ongoing negative stigma associated with acknowledging the onset of CMD and seeking out psychotherapeutic assistance^[1,6]^ seem to precipitate professional rugby players’ reliance on insider (e.g. teammates and medical professionals) support structures during SRI recovery rather than the seeking out or acceptance of external assistance, such as psychotherapists.^[3,6]^ A psychotherapeutic intervention aimed at lessening psychosocial risk associated with the negative effects of SRI should consider the creation of support structures coming from within the rugby environment and hence, possibly minimising risk of stigma towards seeking psychotherapeutic support.

## Psychosocial developmental factors

In addition to the abovementioned example of socio-cultural factors that can affect SRI recovery protocols, age-related psychosocial developmental factors should also be considered. Shinke et al. ^[8]^ in their position stand on athlete’s mental health, performance and development suggested that injured elite athletes experience heightened pressure situations during early adulthood. Early adulthood is a developmental stage wherein individuals are most vulnerable to the onset of psychopathology.^[8,12]^ Furthermore, early adulthood includes individuals seeking out intimacy in both platonic and romantic relationships, the achievement of which can lessen the risk of the development of mental health disorders.^[12]^ It would, therefore, be reasonable to assume that professional rugby players, experiencing SRI during the psychosocial stage of early adulthood would benefit from interventions that considered opportunities for the injured player to form both close relationships and networks of support.

Psychotherapeutic interventions allied to sport rehabilitation processes exist.^[10]^ These interventions include educational interventions, goal setting, imagery for performance, imagery for rehabilitation, self-talk-based interventions, biofeedback, and social support-based interventions.^[10]^ However, it seems as though no psychotherapy intervention currently exists that takes advantage of opportunities presented in team-specific environments for interpersonal learning, relationship building, and real-time skills acquisitions that are documented as being protective factors against the onset of CMD.^[13]^

## Objective

This paper aims to introduce a psychotherapeutic group intervention designed to address negative psychosocial sequelae linked to SRI in professional rugby player cohorts, while also lessening risks associated with the onset of CMD. In order to achieve these aims, the components of the group therapy intervention named the Recovery Mastery Group (RMG) will be discussed and outlined as a Methods section. Following the Methods section, a short case illustration describing the implementation of the RMG within a professional South African rugby union team will be presented. As this paper aims to introduce the RMG, the group therapy’s component parts, as well as their reciprocal relationships are the focus of the case illustration. Individual group members’ biopsychosocial case histories, injury details, and specific SRI reactions are important facets in a discussion around focused therapeutic outcomes; however, they are not the focus of this paper.

## Methods

Literature supporting the implementation of group therapy as a viable, evidence-based practice in addressing and even redressing clinical and sub-clinical mental disorders in general populations is well established.^[13]^ Group therapy processes are often defined by the members that make up the group, as well as the collective aims of the group.^[13]^ Furthermore, it is the group itself that enables therapeutic factors – the group therapist often takes up a facilitator role aimed at encouraging interpersonal and relational processes.^[13]^ This section describes the three therapeutic components of the proposed group therapy intervention, and their integration within the professional SRI population.

### The Recovery Mastery Group (RMG)

The RMG was designed as a time- and cost-efficient,^[13]^ open-ended group psychotherapy process. The RMG serves as a purposeful insider, psychosocial, developmentally appropriate support structure.

Therapeutic time and cost-efficiency are important factors to consider in the professional rugby environment. Time is often taken up by a variety of essential daily conditioning, strategy, training, and recovery tasks. Furthermore, team budgets do not often include psychotherapeutic concessions. Group therapy sessions are frequently regarded as being cost-effective due to the facilitator being able to see many participants at once.^[13]^ Players can attend weekly RMG sessions at their specific clubs during days already allocated to recovery protocols. Additionally, on-site sessions, consistently facilitated by a team psychologist, might create the perception of the group process as coming from insider support rather than potentially threatening outsider ones. This can reduce issues of stigma associated with psychotherapy, while promoting therapeutic adherence and the greater likelihood of effective recovery outcomes.^[14]^

The RMG employs the integration of Yalom’s^[13]^ therapeutic change mechanisms, a stage-wise WITS trauma framework that serves as a collection of interchangeable guiding principles of trauma recovery^[15]^, as well as guided imagery techniques that have been documented as being effective in promoting injury recovery, general well-being, and sports performance.^[10]^ Regarding integrated psychotherapy, the incorporated framework, change mechanisms, and specific techniques work together to affect change,^[16]^ as is discussed in the case illustration.

### RMG therapeutic factors

Yalom^[13]^ suggested that the efficacy of group therapy is due to present-focused, ‘here and now’ therapeutic change mechanisms that exist within the process/relational aspects of the group itself. Yalom’s change mechanisms include: the instillation of hope, universality, imparting of information, altruism, corrective re-enactment of the primary family group, development of socialising techniques, imitative behaviour, interpersonal learning, group cohesiveness, catharsis, and existential factors.^[13]^

General, positive relational experiences aligned to interpersonal learning, group cohesion, universality, and the instillation of hope can be considered protective factors against the onset of mental health issues during early adulthood.^[12]^ They have also been documented as being supportive elements that might promote injury recovery efficacy in rugby and sporting populations.^[6,7]^

Considering the trauma-related effects of severe rugby injury (e.g. fear and stress reactions), the abovementioned group therapy change mechanisms should be experienced and integrated within an interchangeable stage-wise trauma therapy framework.

### RMG trauma therapy framework

A major factor in addressing experiences of both physiological and psychological trauma is how effectively an individual is able to reconstruct idiosyncratic meaning structures that have been shattered by the traumatic event.^[15]^ The RMG incorporates the South African-developed WITS Trauma Model^[15]^ as a framework for both understanding and ‘working with’ SRI trauma reactions.

The WITS Trauma Model consists of a five-stage interchangeable therapy framework: telling/retelling the trauma story, normalising symptoms, addressing guilt and self-blame, encouraging mastery, and facilitating the creation of meaning. The framework aims at re-attaching meaning to both the traumatic event and the individual’s life, while striving towards trauma mastery.^[15]^

Regarding the RMG, the trauma therapy framework is employed as a set of ‘guiding principles’ from which group therapy change mechanisms might operate. For example, an injured player might feel compelled to tell/retell the story of her/his injury onset within the group, thus providing her/him the opportunity to experience change mechanisms, such as catharsis, universality, and ‘real-time’ interpersonal learning.

Furthermore, the integration of a guided imagery technique at the outset of every session presents the group members with a practical approach to mitigating stress reactions, as well as with a platform from which to share pertinent recovery protocols and experiences.

### RMG guided imagery techniques

Originally, the RMG included a mindfulness awareness technique aimed at promoting stress reduction, pain management, and the development of a strong mind-body connection.^[17]^ However, some group participants experienced this technique as being stress-promoting due to them not being able to retain focused awareness on themselves. It was then decided to employ guided imagery techniques, which is more experience-focused rather than awareness-focused. This change is discussed in the Case illustration below.

The inclusion of guided imagery techniques within the RMG framework aims to address potential negative affective states found within the injured professional rugby player population, while promoting motivation for recovery outcomes.^[10]^ The technique includes individuals being asked to imagine certain scenes and then being guided through a series of visualised experiences. Research on the effects of guided imagery and visualisation techniques in competitive sports suggest that these techniques can promote relaxation, lessening of stress and anxiety, bolstered self-confidence, and improved sports performance. Regarding, sports injury rehabilitation, guided imagery has been shown to assist in muscle relaxation, the reduction of stress hormones, the promotion of recovery motivation, and coping with pain and negative emotions.^[10]^

In summary, the RMG attempts to integrate Yalomian group therapy change mechanisms, the WITS Trauma Model, and guided imagery techniques in order to promote psychosocial SRI recovery in both time- and cost-effective ways.

## Case illustration

The following section illustrates the implementation of the RMG with a South African professional rugby team during 2019. Attention is drawn to examples of how this group’s experiences of SRI psychosocial reactions were addressed via the integration of the overarching RMG trauma recovery framework, RMG therapeutic change mechanisms, and guided imagery techniques.

### Group member recruitment

Eight players were recruited from the team. The ages of the participants ranged from 19 to 32 years old. Members included those players who had experienced an SRI and who medical personnel, including the team sports physician, perceived as showing concerning psychological and behavioural changes during their recovery. Chang et al.^[9]^ suggested that sports physicians and other sports medical personnel are uniquely situated to detect the need for psychological support in the elite athlete population. Differences in age, experience, and injury recovery stages promoted a heterogeneous group makeup that encouraged cross-experiential, interpersonal learning.^[13]^

### Group structure

Due to the open-ended structure of the group, two newly injured players entered into the RMG during the 12 sessions and one player, who had recovered, exited the group after Session 10.

Each session began with an introduction which included group rules and the necessity for confidentiality. The group then took part in a five- to seven-minute guided imagery exercise. The group experience was thereafter unstructured until the final five minutes comprising the closing ritual (in this case, selecting two group members who would supply the RMG with coffee the following week). Group sessions were 60 minutes long and facilitated during a time in the training day when no other team activities had been prepared. Hence, both newly injured and ‘returning to play’ group members would be able to attend.

### Experiences of RMG therapeutic factors

The following case information, including pertinent SRI group psychosocial themes and group member feedback, was gleaned from facilitator process notes. Presented case information was selected by the authors perceived as being relevant in outlining the intended reciprocal relationship between the aforementioned RMG therapeutic factors. Paraphrased vignettes, intended to protect the identities of group members, are employed as examples of group member experiences.

Themes of negative psychosocial sequelae linked to SRI included group members’ experiences of disbelief at the onset of the SRI, feelings of isolation, fear related to recovery and future performance, socially avoidant behaviours, experiences of both diminished hope and self-confidence, as well as individual difficulties in understanding emotional reactions to injury. These factors were consistent with those found in the literature exploring psychosocial sequelae of SRI.^[2,3,4,5,6]^ Examples of how the integration of RMG therapeutic factors, mindfulness and guided imagery techniques, as well as how confrontation and relating in the group process addressed group specific themes of SRI reactions, are presented below.

### Integration of RMG therapeutic factors

Firstly, the RMG afforded group members opportunities to normalise feelings of isolation, fear, and diminished hope by allowing them to recite their experiences in the group setting. The telling and retelling of the trauma experience and the normalisation of psychological trauma symptoms have been documented as being effective therapeutic tools in processing trauma.^[15]^

For example, group member B, disappointed at the timing of his injury (in his first game back after being previously injured), consistently steered the group conversations towards his experience of injury onset. He seemed to need to tell and retell the story of his re-injury experience.

- ‘… I mean, it happened … again … I remember when I felt it go – whack – I knew it was gone again … all I could think about was, ‘… not again, not again … what’s my dad going to say? How am I going to go through another six months, sitting at home with my parents?! … I know I’ve said this before, it’s just … you know’

Secondly, the RMG appeared to address the aforementioned SRI psychosocial themes via the integration of interpersonal learning and experiences of universality: two group therapy change mechanisms that have been documented as being useful in addressing an array of negative psychosocial symptoms, including trauma and existential issues.^[13,14]^

On one occasion, group member C mentioned that he too thought of his mother and sister at the time of his injury and that he would often repeat the injury experience in his mind. The group setting allowed for the telling and retelling of the injury stories, while group members experienced the normalisation of trauma symptoms via group interpersonal learning.

- ‘… I remember thinking, … “this could be bad … what’s going to happen now, I’m in my last year of contract and my mother needs the money!” … I mean, I still think about what happened, even though I know I’ll get better, I still go over it in my head … its irritating … maybe its normal … I mean, thinking about what happened all the time’

Group member A (an experienced player) commented on the exchange between B and C. He suggested that he too experienced recurring frustrations around how his injury had occurred but he reassured B and C that these thoughts would eventually dissipate and that they would begin to focus on using their recovery processes to become better players, overall.

- ‘… its annoying to keep thinking about these things [injury onset] but I promise, eventually, it gets better … eventually you start to think, ‘… this is time I have to make myself better!’ … we hardly get time to improve on things during the season … so you can use this time, now … it gets better, I promise’.

Group member A had inadvertently instilled hope, outlined a universal theme of shared injury experiences, and presented an opportunity for interpersonal learning. The above interaction took place within the framework of the telling/retelling of the injury/trauma story, normalising trauma symptoms, and developing an opportunity for mastery of the trauma experience. The group structure seemed to create a platform from which injured RMG members could experience connectedness rather than isolation, as well as the instillation of hope for both future recovery and performance – positive psychosocial responses to SRI that can promote recovery efficacy.^[6,7]^ This would have been difficult to achieve outside of a group session.

During group member feedback sessions, A, B, and C mentioned that the RMG sessions had assisted them in creating experiences of ‘friendship’, ‘connection’, and ‘meaning through learning from each other’.

### Mindfulness and guided imagery techniques

A mindfulness technique comprising becoming aware of one’s breath and a gradual, systematic non-judgmental awareness of each major body part^[17]^ was initially employed at the beginning of the first three RMG sessions. Some group members suggested that they experienced difficulties in maintaining a detached awareness of their body parts, due to physical pain and the need to perform.

Group member D indicated that he struggled to maintain an awareness of his injured body part without judging himself in a harsh and angry way. He expressed to the group that he felt like he was not ‘doing it right!’. D seemed to experience behavioural stress in his need to ‘perform’ in the ‘detached’ mindfulness space.

- ‘… can you guys do it? … I can’t focus … I keep thinking of my injury, and I’m angry … you say “be aware of your neck” and I just go straight to my knee! …, that was hard! …’

Group member E, however, declared that he found the mindfulness exercise incredibly relaxing, but, that he had meditated previously. He proposed that those group members who found it difficult to maintain a detached awareness of themselves potentially lacked experience in meditation and that they would eventually learn to feel relaxed if they continued to practice meditation.

- ‘I actually feel super relaxed! … I’ve done this before, though … I learnt to meditate a while back when I was looking for things to help me relax … it takes practice though … you’ll get it … the trick is to not try too hard. Sounds strange but it’s true …’

Due to the discrepancy in expressed experiences related to the mindfulness practice, it was decided to shift from an awareness experience to a guided imagery technique for the next session.

The guided imagery exercise comprised the facilitator narrating a ‘mind journey’ during which the group members were asked to participate in specific imagery and ‘feeling’ exercises. Guided imagery techniques such as this one are frequently employed to increase feelings of relaxation, and decrease experiences of stress and anxiety.^[10]^

After the application of the guided imagery technique, Group member D exclaimed that he felt that the experience was very powerful. He suggested that he preferred this technique to the previous week’s one.

- ‘… that was powerful! I literally felt like I was a tree with strong roots!’

According to RMG member feedback sessions, differences exist in how severely injured rugby players experience various mindfulness and guided imagery practices. According to this case illustration, guided imagery techniques were preferred by the group, as a whole, when compared with the mindfulness practice.

### Confrontation and relating in the group process

Beginning the group sessions with a guided imagery practice often encouraged group discussions around how members experienced this practice. Frequently, these discussions would lead to session material and group processes that could be explored. For example, during the abovementioned session when Group member D expressed his difficulty in maintaining awareness, the facilitator asked which other group members had similar reactions. The facilitator noticed that Group member F, a junior player, looked as if he want to add to the discussion but would then sit back in his chair, letting other members speak. The facilitator brought the group’s attention to F potentially wanting to add something. Group member G, a friend of F commented, while laughing and in a ‘bantering’ manner, that F ‘never speaks his mind’.

- ‘… *F* doesn’t say much! He’s quiet, that’s his thing … and he’s a scrumhalf!’

F had struggled during his recovery process to communicate recovery information to the medical team. He would often not say that he was experiencing fatigue or pain. He would then avoid certain conditioning sessions in favour of rest or physiotherapy without communicating his decision to the parties involved. This pattern was negatively affecting his relationships with the medical personnel and the coaches. Group member A joined in on the banter and cajoled F into ‘speaking up’.

- ‘… the guys are teasing you hey! You better say something … they not going to stop hassling you now!’

F eventually commented that he also struggled with the mindfulness technique. The facilitator asked F what he thought of the banter that was directed towards him. F suggested that he was a junior player and that he wanted to be respectful to the seniors in the team but that the banter was fine.

- ‘I don’t want to say too much … I’m a junior and it’s important to be respectful … I don’t mind being teased, its actually nice … it’s nice that we [Juniors] can talk like this with guys like *A* who have been there [experienced players]’

Although this group experience was somewhat confrontational for F, he seemed to feel supported by the other ‘bantering’ members. The facilitator used the discussion to broach the topic of communication. Group member A reiterated the importance of being able to confront teammates and even coaches, at times. F stated that he struggled to talk to coaches about how his recovery was going for fear of them seeing him as weak or lazy.

- ‘… I feel like I’m lucky to be here … if I’m tired or sore, I don’t want the coaches to know … they’ll think that I’m trying to get out of stuff or that I shouldn’t be here [contracted]’

The group discussed F’s assumptions around what would happen if he communicated openly to coaches. There was a consensus that it was vitally important to speak to coaches and other personnel regarding recovery experiences and even when returning to play. The following RMG sessions involved group members continuing to express ‘banter’ towards F. The facilitator noted that F began to engage with the group more than previously. Interestingly, during a medical meeting, one of the medical personnel team commented that F had spoken to him about his recovery protocols and that F had asked him if they could change some of the conditioning routines to ones that F felt were more beneficial to him.

The abovementioned process seemed to address issues of diminished self-confidence, communication, and avoidant behaviours via real time confrontation within the RMG. Group members were able to relate to each other between junior and senior hierarchies, while learning skills that were then applied to situations outside of the group context. The translation of in-group relational processes to daily life situations is regarded by Yalom as an indication of group therapy efficacy.^[13]^

In summary, the implementation of the RMG within this specific population seemed to promote desirable individual experiences of telling and retelling the injury story, the normalisation of trauma symptoms, interpersonal learning, experiences of universality, and the instillation of hope. Guided imagery practices reportedly assisted members with stress reduction, while supportive confrontations and inter-member relating assisted members in learning communication skills. Generally, group members reported experiencing increases of general feelings of empowerment by the end of the RMG process. The RMG psychotherapeutic factors mentioned have been proven to mitigate the negative effects of trauma, protect against psychological vulnerability and, in some cases, promote effective injury recovery.^[1,3,4,10]^

### Limitations

This paper is intended to introduce the RMG as a potentially effective group psychotherapeutic intervention aimed at addressing negative psychosocial sequelae linked to SRI in professional rugby player cohorts. However, there are various limitations regarding its presentation.

- Firstly, the case illustration was drawn from the group facilitator’s process notes and contextualised in terms of existing group and individualised psychotherapy interventions. No quantitative data referring to therapeutic efficacy exists.
- Secondly, individual group member data, including age, injury type, recovery stage, and general biopsychosocial case histories were not included in this paper. General therapeutic outcomes were presented as being allied to overarching RMG processes rather than to specific individual factors.
- Thirdly, and due to limited individual biopsychosocial data, reasons for the outlined discrepancy between the inclusion of guided imagery practices as opposed to mindfulness techniques is not clear.
- Finally, interpretations are subjective and, therefore, not necessarily generalisable.

### Future research

In light of the outlined limitations of this paper, further research should include both quantitative and qualitative methods that could elucidate which specific RMG factors produce specific outcomes. Furthermore, studies focused on the discussed discrepancy between guided imagery practices and mindfulness techniques within the SRI population could add to the literature on this topic. Finally, research aimed at understanding differences between the effectiveness of ‘insider’ vs ‘outsider’ support structures could yield valuable data for validating RMG processes.

## Conclusion

This paper introduced the concept of a time- and cost-effective group psychotherapeutic intervention aimed at addressing negative psychosocial effects of SRI and lessening the risk of the onset of CMD in the professional rugby player population. Documented negative psychosocial reactions to SRI and recently documented levels of CMD within the professional rugby population have highlighted the need for specific ‘insider’, consistent, psychosocial support structures within professional rugby. The RMG takes advantage of the somewhat unique professional rugby environment in order to create an ‘insider’ support structure focused on developmentally appropriate relationship building and interpersonal learning. Although more research on the efficacy of specific RMG factors is needed in order to validate the intervention, the authors of this paper would recommend the RMG as an effective therapeutic intervention for this specific population.
